# Influence of curing mode and aging on the bonding performance of universal adhesives in coronal and root dentin

**DOI:** 10.1186/s12903-024-04879-2

**Published:** 2024-10-05

**Authors:** Hoda Saleh Ismail, Ashraf Ibrahim Ali, Mohamed Elshirbeny Elawsya

**Affiliations:** Conservative Dentistry Department, Faculty of Dentistry, Mansoura University, Algomhoria Street, PO Box 35516, Mansoura, Aldakhlia, Egypt

**Keywords:** Bond strength, Deep margin elevation, Self-cure adhesives, Universal adhesives

## Abstract

**Background:**

This study aims to evaluate the microtensile bond strength (µTBS) of different resin composite restorations bonded to mid-coronal dentin and proximal root dentin using light-cured, chemical-cured, and dual-cured adhesives immediately and after aging. Nanoleakage and degree of cure were also assessed.

**Methods:**

Eighty-four molars were divided into mid-coronal dentin and proximal root dentin. Each group was further subdivided into three subgroups based on the restorative systems used, which involved the utilization of light-cured, chemical-cured, and dual-cured adhesives. Half of the specimens underwent µTBS testing after 24 h, while the other half after aging. Representative specimens were analyzed for nanoleakage. The degree of cure of the tested adhesive systems was also assessed.

**Results:**

Aging showed a significant negative effect on µTBS results and led to increased nanoleakage (*p* < 0.001). Furthermore, in all subgroups, the µTBS values of proximal root dentin were lower compared to mid-coronal dentin, except in the aged subgroup for the system utilizing the dual-cured adhesive. The restorative systems with chemical and dual-cured adhesives demonstrated comparable bonding properties. However, the system with the light-cured adhesive exhibited the worst bonding properties after aging when bonded to proximal root dentin and cured at a large distance (*p* < 0.05).

**Conclusions:**

All tested restorative systems were negatively affected by aging, and the regional dentin had variable effects on the bonding properties. Clinicians should exercise caution when using the tested light-cured adhesive in areas where the curing distance exceeds 3 mm.

## Introduction

Contemporary adhesive technology and resin composite materials allow for repairing extensively damaged teeth through directly placed resin composite restorations [1]. For cavities with subgingival margins, the usual method is to use a direct restoration as a base, elevating the cavity margin to a supragingival location [2]. This technique, known as proximal box elevation or the open sandwich technique, has been performed using both glass ionomer-based and resin-based materials [3], however, resin composites are recommended due to their superior properties when bonded with deep cervical margins including wear resistance, elastic modulus, flexural strength, and hardness [3]. Resin composites are commonly chosen due to their compatibility with epithelial tissues and reduced likelihood of periodontopathic bacterial attachment [4]. A recent comprehensive analysis suggested that when restoring proximal cavities with dentin/cementum gingival margins, it is advisable to opt for resin composites rather than the open sandwich technique involving resin-modified glass ionomer [5].

A new adhesive system called “universal” has been introduced in recent years [6], which can be applied to both dentin and enamel using etch and rinse, self-etching, or selective-etching modes depending on the clinical situation [6]. However, simplified light-cured adhesives may not be suitable for use with self-cure and dual resin composites due to incompatibility between the acidic monomers in the adhesive and the tertiary amines used as co-initiators in the composite materials [7]. To address this issue, dual-cured and chemical-cured universal adhesives have been developed that are fully compatible with all types of methacrylate-based composites without requiring an additional activator for dual-curing [8]. The bonding effectiveness of these adhesives is not compromised in the absence of light irradiation [8] making them suitable for use in situations where there may be insufficient light curing, such as when cementing endodontic posts or in deep areas of the cavity [7].

Obtaining satisfactory polymerization is of utmost importance, and one crucial factor to consider is maintaining the correct distance between the light guide tip and the resin composite [9]. However, achieving this optimal distance can be difficult to control. Several factors come into play, including the degree of caries advancement, the size of the cavity, and its location [10, 11]. Light dispersion from the curing unit intensifies when the distance exceeds 2 mm, making optimal polymerization difficult to achieve [10]. Insufficient polymerization is a potential problem in deep cavities of Class I and Class II due to the considerable distance between the light curing tip and the initial resin composite layer [12]. As a result, there is a risk of incomplete solidification of the material in these specific regions [10]. This issue is especially noteworthy in preparations involving deep proximal boxes where the margins primarily involve cementum and dentin presenting a greater challenge for achieving successful bonding compared to proximal gingival margins that still have a layer of cervical enamel intact [5].

In the case of deep proximal margins in Class II cavity restorations, it is a standard procedure to carry out supplementary photo-polymerization from both the buccal and lingual aspects. This is done after removing the matrix-band to address the greater gap between the tip of the light-curing device and the properly positioned composite layer at the gingival margin [13]. However, using a light-cured adhesive with deep subgingival margins carries a potential risk of incomplete curing. This is because the additional curing performed through the embrasures, after matrix-band removal, would be positioned more occlusal than the level of the subgingival proximal margins. As a result, this scenario prompts a significant inquiry about the potential influence of the curing method employed by the adhesive in deep proximal cervical margins on the long-term durability of the restoration.

The strength of the adhesive bond is directly influenced by various structural elements and characteristics of dentin, such as the flow of pulpal fluid and dentin permeability [14]. Predicting the longevity of the bond between adhesive resin and dentin is challenging due to the diverse morphological and physiological variations found in human dentin [14]. The orientation of dentinal tubules is known to significantly affect the formation of the hybrid layer, especially with etch-and-rinse adhesives [15]. Although differences in dentin wetness across regions have been noted, the effect of dentin location on bonding was variable and material-dependent [16]. However, depth may have acted as a confounding variable [16]. Previous studies have shown that resin-dentin bond strengths remain consistent across flat occlusal surfaces without significant variations based on specific regions [17, 18]. Nonetheless, degradation in bonding has been observed in peripheral specimens, particularly in specimens lacking outer enamel [17]. This raises the question of whether regional dentin differences could affect the overall durability of the restoration, particularly in cases involving extended curing distances in deep proximal cervical margins.

Based on the previous data, this study aims to evaluate and compare the µTBS of various resin composite restorations bonded to both mid-coronal dentin and proximal root dentin using light-cured, chemical-cured, and dual-cured adhesives. The tested adhesives were cured at a distance comparable to the clinical situation immediately and after aging. Additionally, nanoleakage at the interfaces of the tested adhesives with both mid-coronal and proximal root dentin was examined, both immediately and after aging. The immediate degree of conversion of the adhesives was also calculated. The study investigated the validity of four null hypotheses: (1) the restorative system type would not significantly affect µTBS and nanoleakage when bonded to mid-coronal dentin and proximal root dentin, both immediately and after aging; (2) water storage followed by thermocycling would not significantly affect µTBS and nanoleakage for various restorative systems bonded to mid-coronal dentin and proximal root dentin; (3) the regional dentin type (mid-coronal dentin or proximal root dentin) would not significantly affect µTBS and nanoleakage for different restorative systems, regardless of the aging process; and (4) there would be no correlation between µTBS and nanoleakage data.

## Materials and methods

### Materials used

This study examined three different restorative systems currently available in the market. The systems consisted of: (1) a light-cured universal adhesive (Tetric N Universal; Ivoclar Vivadent, Amherst, NY, USA) combined with a nanohybrid resin composite material (Tetric N-Ceram; Ivoclar Vivadent), (2) a chemical-cured universal adhesive (Palfique Universal Bond; Tokuyama Dental, Tokyo, Japan) paired with a submicron filled resin composite (PALFIQUE LX5, Tokuyama Dental), and (3) a dual-cured universal adhesive (Futurabond U; VOCO, Cuxhaven, Germany) used in conjunction with a nanohybrid resin composite (Grandio; VOCO). For a comprehensive overview of the materials used in the study, please refer to Table 1.

**Table 1:** Restorative systems used in the study

Restorative System	Material	Shade	pH	Type	Manufacturer	Composition	Application technique	Lot number
System with the light-cured adhesive	Tetric N‑Bond Universal		2.5	Light-cured universal adhesive	Ivoclar Vivadent, Amherst, NY, USA	10‑MDP, MCAP, HEMA, Bis‑GMA, D3MA, ethanol, highly dispersed silicon dioxide, water, initiators, and stabilizers	1. Apply bond with rubbing action for 20 s. 2. Dry for 5 s with oil-free gentle air stream. 3. Light cure for 10 s.	Z04LY6
	Tetric N-Ceram	A2		Nanohybrid resin composite	Ivoclar Vivadent	Bis-GMA, Bis-EMA, TEGDMA, Barium glass, ytterbium difluoride. Filler Volume %: 56		Z03Z6k
System with the chemical-cured adhesive	Palfique Universal Bond (Tokuyama Universal Bond)		2.2	Chemical-cured universal adhesive	Tokuyama Dental, Tokyo, Japan	Liquid A: 3D-SR monomer, MTU-6, HEMA, Bis-GMA, TEGDMA, acetone. Liquid B: Silane coupling agent (γMPTES), borate catalyst, peroxide, acetone, isopropyl alcohol, water	1. Mix one drop of each liquid A and B thoroughly then apply it. 2. Air-dry with mild air within 30 s after application until the runny adhesive stays in the same position without any movement.	161E01
	PALFIQUE LX5	A2		Submicron filled resin composite	Tokuyama Dental	Bis-GMA, TEGDMA, silica-zirconia filler and composite filler. Filler Volume %: 71		E8464
System with the dual-cured adhesive	Futurabond U Single dose		2.3	Dual-cured universal adhesive	VOCO, Cuxhaven, Germany	Liquid 1: HEMA, Bis-GMA, HEDMA, acidic adhesive phosphate monomer, Urethane dimethacrylate, catalyst. Liquid 2: ethanol, Initiator, catalyst.	1. Mix and stir thoroughly the two liquids 2. Apply the adhesive with rubbing motion for 20 s. 3. Air-dry with a gentle stream of air over the liquid for about 5 s until it no longer moves. 4. Light cure for 10 s.	2309170
	Grandio	A2		Nanohybrid resin composite	VOCO	Bis-GMA, TEGDMA, silicon dioxide nanofillers and glass ceramic microfillers. Filler Volume %: 71.4		2250322

Abbreviations 10-MDP: 10-methacryloyloxydecyl dihydrogen phosphate, MCAP: methacrylated carboxylic acid polymer, HEMA: hydroxyethyl methacrylate, Bis-GMA: bisphenol A-glycidyl methacrylate, D3MA: decandiol dimethacrylate, Bis-EMA: bisphenol-A polyethylene glycol diether dimethacrylate, TEGDMA: triethyleneglycol dimethacrylate, 3D-SR: three-dimensional self-reinforcing, MTU-6: 6-methacryloyloxyhexyl 2-thiouracil 5-carboxylate, γ-MPTES: γ-methacryloxypropyl triethoxy silane, HEDMA: hexamethylene dimethacrylate

## Sample size calculation

Before commencing the study, the required sample size for µTBS testing was calculated using GPower software (Ver. 3.1.9.7; GPower, Kiel, Germany). The calculation was based on a similar study design from a previous study [19]. The study compared the immediate aged µTBS values of single-layer data of a single bond universal. The following parameters were considered: a two-tailed test, an effect size of 1.59, a significance level (α) of 0.05, 80% power, and an allocation ratio of 1. The calculated sample size per subgroup was 7.

### Microtensile bond strength testing

#### Selection and preparation of teeth

A total of 84 human upper molars were selected for µTBS analysis. These teeth were extracted due to periodontal disease and exhibited comparable dimensions (The included teeth for µTBS exhibited mesiodistal dimensions of 9–9.5 mm and buccopalatal dimensions of 10–10.5 mm). Each tooth underwent a thorough examination using a stereomicroscope (Olympus model SZ-PT, Tokyo, Japan) to ensure the absence of caries and cracks. Soft tissue and calculus deposits were meticulously removed by using an ultrasonic scaler during the teeth cleaning process. Subsequently, the teeth were stored in a 0.5% Chloramine T solution until ready for use. All teeth were utilized within six months of extraction. Patients were informed about using their teeth in the study and presenting written informed consent. The Institutional Review Board (IRB; M0108023CD) approved the collection of teeth for this study.

The tooth roots were firmly held vertically using self-curing acrylic blocks to simplify the preparation and restoration procedures. These blocks were placed at a specific depth of 3.0 mm below the CEJ. To ensure accurate positioning of each tooth, a specifically designed jig device was used during the fixation process. The teeth were then randomly divided into two groups, each consisting of 42 teeth based on the region of dentin being tested.

In the first group, the occlusal surfaces of the teeth were cut parallel to the occlusal table and perpendicular to the tooth’s long axis at the midpoint of the crown. A slow-speed diamond saw (Isomet 4000, Buehler Ltd., Lake Bluff, IL, USA) with a coolant was used for this sectioning technique. It resulted in exposed flat mid-coronal dentin surfaces without any damage to the pulp chamber. In the second group, the teeth were horizontally sectioned 1 mm below the CEJ. In both groups, a standardized smear layer was created by using 600-grit silicon carbide paper (Microcut, Buehler Ltd.) with a continuous water flow for 60 s [20].

### Experimental design and restorative procedures

After tooth preparation, the teeth were rinsed with water and dried. Each group was randomly divided into three subgroups, with 14 teeth in each subgroup.

Following the creation of the smear layer, the dentin surfaces were carefully rinsed and dried, avoiding excessive drying. Subsequently, the appropriate adhesive specific to each restorative subgroup was applied to all the dentin surfaces. The adhesive was then air-thinned and light-cured (For the light and dual-cured adhesives) according to the manufacturer’s instructions, as detailed in Table 1.

During the photo-polymerization process, two different distances were maintained between the tip of the light-curing device and the dentin surface. For the mid-coronal dentin groups, the distance was set at 3 mm, while for the proximal root dentin groups, the distance was increased to 7 mm. Opaque acrylic plastic rings with heights corresponding to the respective distances were used to ensure consistent distances [21]. Each ring, with a 10 mm opening, was carefully positioned to make light contact with a 0.5 mm enamel rim along the top mid-coronal dentin/adhesive surface on either the buccal or palatal sides of each tooth specimen (for mid-coronal dentin specimens). This was accomplished while ensuring central alignment with the long axis of the crown. For proximal root dentin specimens, which had dimensions less than 10 mm, the ring was placed so that the border of the opening was flush with the dentin/adhesive surface level, maintaining centralization with the long axis of the crown. Subsequently, the adhesive was polymerized with the tip of the light-curing device positioned directly over the ring. To initiate the curing process, an LED curing light (Elipar Deep Cure; 3 M ESPE, St. Paul, MN, USA) with a power intensity of 1200 mW/cm^2^ was used. The curing procedure was carefully monitored at regular intervals with a radiometer (Demetron L.E.D. Radiometer, Kerr Corp., Orange, CA, USA) to measure the effectiveness of the curing after every set of five specimens.

The specimens were restored by applying a 4.0 mm-thick layer from resin composite specific to each subgroup. The composite material was applied in two horizontal increments with each increment being 2 mm thick. A gold-plated composite placement instrument (Zeffiro, Lascod SpA, Italy) was used to adapt the composite and ensure proper placement. The curing process for each increment was carried out separately following the manufacturer’s recommendations by light curing from the occlusal surface. The curing procedures were performed using the same LED device utilized for the polymerization of both light- and dual-cured adhesives. Additionally, regular monitoring of the curing procedures was conducted using the radiometer mentioned previously.

To facilitate the restoration process, a contoured Tofflemire matrix-band was placed around each tooth. After removing the matrix-band, all restorations underwent an additional round of light curing from the side for 20 s. Following this, the specimens were stored in distilled water at 37 ± 1 °C in an incubator for 24 h. All tooth preparation and restoration procedures were performed by a single operator throughout the study. The operator used magnifying loupes (×4 loupes, Amtech, Wenzhou, China) and LED headlight illumination (HLP05, Amtech).

### Artificial aging

The specimens in each subgroup were randomly divided based on the aging condition with seven specimens in each condition. The specimens in the first aging condition were immediately tested after being incubated in sterile water at 37 ± 1 °C for 24 h. The specimens in the second aging condition underwent an additional aging process. They were stored in sterile water for 6 months at 37 ± 1 °C in an incubator, with weekly changes of water, and then subjected to thermocycling (SD Mechatronik Thermocycler, Germany) to simulate approximately 6 months of clinical service [22, 23]. The thermocycling consisted of 5,000 cycles.

During thermocycling, the specimens experienced temperature fluctuations between 5 °C and 55 °C, with a tolerance of ± 2 °C. These temperature variations followed the recommendations outlined in ISO 11,405 by the International Standards Organization. The dwell time at each temperature was 25 s, and there was a 5-second transfer time between the two temperature baths [24].

**Fig. 1:**
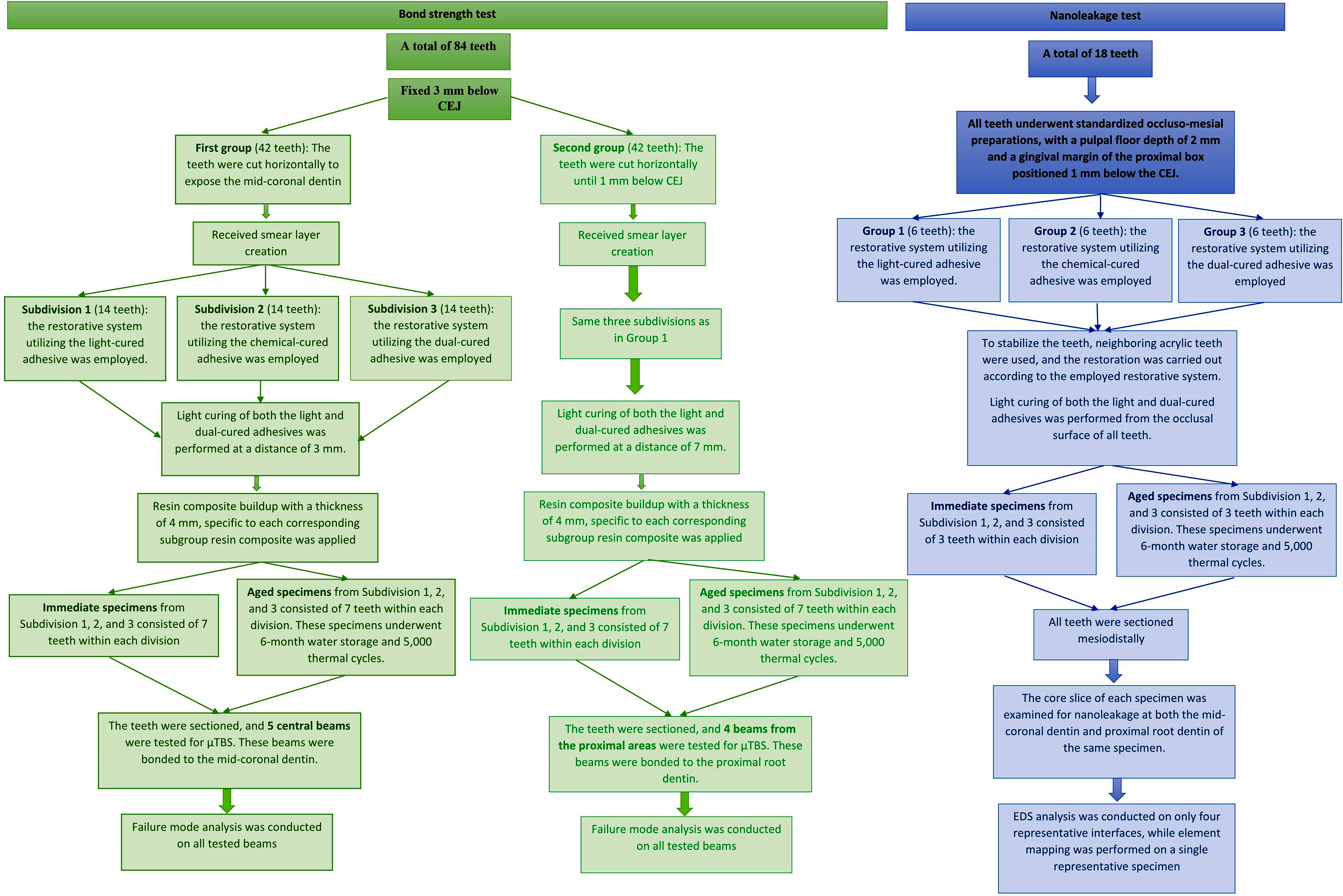
A schematic illustration depicting the experimental grouping and all the steps involved in specimen preparation for the microtensile bond strength and nanoleakage tests in the current study

Upon completing the aging protocol, all specimens were carefully examined under an optical microscope to detect any cracks or damage. To differentiate and track each tooth within the aging condition subgroup, they were assigned a specific color and numbered from 1 to 7. Before sectioning from the occlusal surface for testing purposes, the central area of the resin composites in the mid-coronal dentin groups and the proximal resin composite areas in the proximal root dentin groups were marked. A schematic illustration of the experimental grouping and all the steps involved in specimen preparation for the µTBS test is presented in Fig. 1.

### Specimen preparation

The bonded specimens underwent a transformation process where they were converted into rectangular sticks referred to as beams. This transformation was achieved by cutting the specimens in the mesiodistal and buccolingual directions, perpendicular to the bonded interface. To carry out this sectioning process, a slow-speed diamond saw with water coolant was used. The resulting beams had a cross-sectional area of 1 mm². The top half of the beams consisted of resin composite, while the bottom half consisted of coronal or proximal root dentin.

The width and thickness of each beam were accurately assessed using a digital caliper with a precision of 0.01 mm. In the group of mid-coronal dentin, five central beams were randomly chosen from each specimen for subsequent testing. However, in the group of proximal root dentin, only four beams from the proximal areas were selected for testing purposes.

### Microtensile bond strength evaluation

To attach the beams to the universal testing machine (Instron, Model: 3345, Norwood, MA, USA), Geraldeli’s jig was used. The beams were carefully positioned within the central groove of the jig and were securely fastened at their ends using cyanoacrylate-based glue (Zapit, DVA Inc, Corona, CA, USA). Subsequently, the jig combined with the beams, was connected to the testing machine using a 500 N load cell.

A tensile load was gradually applied at a cross-head speed of 0.5 mm/minute until failure occurred. The resulting bond strength was determined in mega-Pascal units (MPa) using the Bluehill Lite software (Instron, Norwood). After testing, the fragments of the beams were carefully removed from the jig using a scalpel. They were then examined under a stereomicroscope at ×40 magnification (Olympus model SZ-PT) from both sides to identify the specific mode of failure. The failure patterns were categorized as follows: (1) adhesive failure at the interface between the resin and dentin, (2) cohesive failure within the resin composite, (3) cohesive failure within the dentin, or (4) mixed failure involving both adhesive fracture and cohesive failure in the composite, with or without dentin fracture.

Any specimens that experienced failure before the actual testing were counted and documented for each subgroup within each aging condition. However, these particular specimens were not included in the subsequent statistical analysis. All test procedures were conducted by a skilled operator who remained blind to the restorative steps.

### Nanoleakage testing

#### Teeth selection, preparation, fixation, restorative steps, and aging

A total of 18 upper molars were used for this test. The criteria for selecting these teeth were the same as those mentioned for the µTBS evaluation. Additionally, teeth with a mesial occluso-gingival length ranging from 6.5 to 7 mm were carefully chosen. This measurement was obtained from the highest point of the cusp to the CEJ. The highest cusp was adjusted using a water-cooled diamond disc until a consistent distance of 6 mm between the highest cusp and the CEJ was achieved [25].

The complete detailed cavity preparation and restoration steps are described in a previous study [25]. Uniform occluso-mesial preparations were performed on all teeth using a standard approach. A cylindrical medium-grit diamond bur and high-speed handpiece with water coolant were used with the bur being changed every five teeth. The preparations had specific dimensions: a bucco-lingual width of 3 mm and an occlusal depth of 2 mm measured from the cavosurface margin of the cavity (The distance between the adjusted highest cusp to the cavosurface margin was approximately 1 mm).

For the box part, the base had a mesio-distal dimension of 1.5 mm, a bucco-lingual width of 3 mm, and extended 1 mm below the CEJ. Measurements were taken with a periodontal probe. After preparation, cavities were carefully examined for defects. Teeth were then randomly grouped into three sets (*n* = 6) based on the restorative system, with each group identified by a unique color. Following the preparation process, each tooth was stabilized using neighboring acrylic teeth on the mesial and distal sides, aided by condensation silicone impression material (Ghenesyl putty, Lascod, Italy). The detailed procedure for achieving this setup was previously described in another study [26].

Enamel margins were selectively etched with 37% phosphoric acid gel (N-Etch, Ivoclar Vivadent) for 15 s followed by a 15-second water rinse and thorough drying. Adhesive tailored to each restorative group was applied to prepared surfaces following µTBS evaluation steps. Light-cured and dual-cured adhesives were applied and cured from the occlusal surface emulating clinical practice with the curing distance standardized by positioning the light-curing device contacting the highest cusp tip. The complete restoration steps, using the same resin composites tested in the µTBS evaluation, were described in detail in another study [25]. After the restorative procedures were completed, all the teeth were removed from their silicone blocks and placed in distilled water at a temperature of 37 ± 1 °C for 24 h in an incubator.

Following the restorative procedures, the teeth belonging to each restorative system group were randomly divided into two subgroups (*n* = 3). They were then exposed to the same aging conditions mentioned in the µTBS evaluation.

### Sectioning

After each aging condition, all specimens were securely mounted vertically in acrylic blocks. To facilitate further analysis, each tooth was longitudinally sectioned in a mesio-distal direction using a precision cutter (PICO 155, PACE Technologies, AZ, USA) with water coolant assistance. Two cuts were made on each specimen resulting in three slices per specimen. The middle slice had a precisely controlled thickness of 1.0 mm. A schematic illustration of the experimental grouping and all the steps involved in specimen preparation for the nanoleakage test is presented in Fig. 1.

### Nanoleakage evaluation

The core slice from each specimen was used to examine nanoleakage at the bonded interfaces. A solution of ammoniacal silver nitrate was prepared following the instructions of Tay et al. [27]. These slices were immersed in the ammoniacal silver nitrate solution in darkness for a day, were thoroughly rinsed in distilled water, and were placed in a photo-developing solution (Kodak, Rochester, NY, USA) for 8 h under a fluorescent light. The specimens were then rinsed in water for one minute. Afterward, the specimens were progressively polished with wet SiC papers and 1 μm diamond paste using a polishing cloth. Subsequently, they underwent ultrasonic cleaning (XH-E412 ultrasonic cleaner, Zhengzhou Xinghua Dental Equipment Co., Ltd, China), were air-dried, mounted on stubs, and coated with carbon-gold.

The examination of resin-dentin interfaces involved utilizing a scanning electron microscope (SEM; FEI Quanta 3D 200i, Thermo Fisher Scientific Inc., MA, USA) in backscattered mode and energy dispersive x-ray spectroscopy (EDS; Thermo Scientific Pathfinder). Six micrographs were taken of each resin-dentin bonded slice with three focusing on each regional dentin area, at a magnification of ×1,000. The percentage of nanoleakage within the adhesive and hybrid layers in each specimen within every regional dentin area was assessed across all images in a specified area (height × width = 40 × 300 μm) on each image using the software (Image J software version 1.53, National Institutes of Health, Bethesda, MD, USA) by a researcher unaware of the specifics [28]. The values obtained from the same specimen within each regional dentin were separately averaged for statistical assessment. Likewise, the total nanoleakage percentages within each interface were averaged for statistical purposes considering the three specimens within each group and their respective aging conditions.

EDS assessments were performed on four distinct interfaces within four specimens from the three examined restorative systems. These evaluations were conducted in designated interface areas to determine the elemental makeup of the entire region. Additionally, element mapping was carried out in a specific region within the mid-coronal immediate interface of the system with the dual-cured adhesive to identify elements like silver, calcium, and silicon.

### Degree of conversion evaluation for the tested adhesives

A silicone matrix, measuring 4 mm in diameter and 1 mm in thickness, was securely fixed onto a Mylar strip (Quantum mylar, 10 mm width, Medi-Dent, Australia), which was placed on top of a glass slide (Sail brand microscopic slide with a thickness of 1–1.2 mm, China). This assembly was placed inside a dark chamber with the bottom side open. Opaque acrylic plastic cylinders with heights of 2 mm and 6 mm and a diameter of 10 mm were attached to the bottom side of the glass slides for the specimens of the light- and dual-cured adhesives. The silicone matrix was filled with the adhesive system using a pipette. Ten specimens were created for the light-cured and dual-cured adhesives, while five specimens were made for the chemical-cured adhesive. The light-cured and dual-cured adhesives were air-thinned and light-cured at distances of either 3–7 mm (Height of the cylinder + 1 mm for the glass slide thickness) according to the manufacturer’s instructions with the light curing tip placed at the opening of the opaque acrylic plastic rings side (*n* = 5 for each curing distance). The chemical-cured adhesive was thinned with air and left to chemically polymerize after mixing. All adhesive specimens were stored in a dark, dry container at 37 ± 1 °C in an incubator for 24 h before testing. The degree of conversion of the adhesive layer in contact with the Mylar strip was evaluated.

Raman spectra of all specimens were recorded using the WiTec Raman imaging microscope (alpha 300R, Germany) coupled with a 532 nm laser line and a ×50 objective. The CCD spectrograph was calibrated for zero, and the signal was enhanced using a silicon wafer. The spectra were collected with the following parameters: laser power of 20 mW, integration time of 20s with 10 accumulations, 600 g/mm BLZ = 500 nm grating, and a ×20 objective (Zeiss EC Epiplan 20x/0.4, working distance 3.3 mm). The initial spectrum of the unpolymerized adhesive system specimen was recorded and used as a reference. All spectra were acquired in the wavelength range between 1000 and 1700 cm^− 1^.

The calculation of the double-bond content ratio between the monomer and polymer in the adhesive was performed using the subsequent formula:

DC (%) = 1 – [R(cured) / R(uncured)] × 100,

Where “R” represents the ratio of intensities between the aliphatic and aromatic peaks at 1639 cm^− 1^ and 1609 cm^− 1^ in both the cured and uncured adhesives. The average value of the measurements obtained from the five specimens of each adhesive was used for statistical analysis.

### Statistical analysis

The bond strength values for each tooth in every subgroup were determined by averaging the mean µTBS values (in MPa) of five beams for the mid-coronal dentin group and four beams for the proximal root dentin group per tooth. Each tooth served as the statistical unit. Statistical analysis was conducted using SPSS (version 20, IBM, Chicago, IL, USA). The µTBS exhibited a normal distribution according to the Kolmogorov-Smirnov test allowing for the application of parametric tests for comparing the study groups. A three-way analysis of variance (ANOVA) was performed to assess the effect of the study variables (restorative system type, aging condition, and regional dentin) and their interactions on bond strength. Post-hoc analysis was carried out with Bonferroni adjustment (at *p* < 0.05). Failure-type data were statistically evaluated using cross tabs and the Chi-Square test to compare failure pattern distributions across different study variables. Pre-test failure data were statistically analyzed for the restorative system type using one-way ANOVA, while independent t-tests were employed for the other two variables.

The nanoleakage values (expressed as a percentage) were analyzed using the Kruskal-Wallis test to determine the effect of each study variable on the nanoleakage results. The Mann-Whitney *U* test was used in the existence of a significant effect. The Spearman correlation coefficient was calculated to determine the correlation between µTBS and nanoleakage values. The degree of conversion data was analyzed with a one-way ANOVA test, followed by post-hoc testing with Bonferroni adjustment (at *p* < 0.05).

## Results

### Microtensile bond strength results

Table 2 presents the mean µTBS values and standard deviations for all studied subgroups. Three-way ANOVA analysis confirmed that the restorative system type variable had no significant effect on bond strength values (*p* = 0.18), while aging and regional dentin type had a significant effect on the results (*p* < 0.001). All interactions between variables were significant, except the interaction between aging and regional dentin type (restorative system and aging interaction: *p* = 0.03, restorative system and regional dentin type: *p* = 0.005, aging and regional dentin type: *p* = 0.174, restorative system, aging, and regional dentin type: *p* = 0.014).

**Table 2:** Mean ± SD (95% confidence interval lower bound-upper bound) of microtensile bond strength values in MPa among tested restorative systems with both mid-coronal dentin and proximal root dentin immediately and after aging

Restorative systems	Regional dentin	Immediate	Aged
System with the light-cured adhesive	Mid-coronal dentin	34.29 ± 6.22 (28.54–40.05)^a^	28.43 ± 6.56 (22.36–34.50)^b^
	Proximal root dentin	31.24 ± 5.53 (26.12–36.35)^b^	14.05 ± 2.37 (11.86–16.24)^e^
System with the chemical-cured adhesive	Mid-coronal dentin	30.24 ± 3.63 (26.88–33.59)^b^	24.18 ± 3.23 (21.19–27.16)^c^
	Proximal root dentin	28.25 ± 6.36 (22.37–34.13)^b^	20.27 ± 4.34 (16.25–24.29)^d^
System with the dual-cured adhesive	Mid-coronal dentin	29.13 ± 5.67 (23.88–34.37)^b^	22.38 ± 3.91 (18.76–25.99)^cd^
	Proximal root dentin	27.31 ± 2.83 (24.68–29.93)^b^	23.51 ± 5.79 (18.15–28.86)^c^

Groups identified with the same superscripted lower case letters are not significantly different among each other. (*p* < 0.05)

**Table 3:** Failure modes distribution and pre-test failure in percentages (Number of beams) among the tested restorative systems with both mid-coronal dentin and proximal root dentin immediately and after aging

Restorative systems	Regional dentin	Immediate					Aged				
		Adhesive	Cohesive in composite	Cohesive in dentin	Mixed	Pre-test failure	Adhesive	Cohesive in composite	Cohesive in dentin	Mixed	Pre-test failure
System with the light-cured adhesive	Mid-coronal dentin	74.30% (26)	8.6% (3)	0% (0)	17.1% (6)	0% (0)	71.4% (25)	0% (0)	0% (0)	25.7% (9)	2.9% (1)
	Proximal root dentin	60.70% (17)	0% (0)	7.1% (2)	21.4% (6)	10.7% (3)	71.4% (20)	0% (0)	0% (0)	14.3% (4)	14.3% (4)
System with the chemical-cured adhesive	Mid-coronal dentin	62.9% (22)	8.6% (3)	0% (0)	20% (7)	8.6% (3)	80% (28)	0% (0)	0% (0)	8.6% (3)	11.4% (4)
	Proximal root dentin	78.6% (22)	0% (0)	0% (0)	14.3% (4)	7.1% (2)	64.3% (18)	0% (0)	0% (0)	25% (7)	10.7% (3)
System with the dual-cured adhesive	Mid-coronal dentin	65.7% (23)	0% (0)	0% (0)	28.6% (10)	5.7% (2)	65.7% (23)	0% (0)	0% (0)	25.7% (9)	8.6% (3)
	Proximal root dentin	57.1% (16)	10.7% (3)	0% (0)	28.6% (8)	3.6% (1)	67.9% (19)	0% (0)	0% (0)	25% (7)	7.1% (2)

Within the mid-coronal dentin group, the restorative system with the light-cured adhesive exhibited statistically higher bond strength compared to the other two systems (*p* < 0.05), which had comparable bond strength values, irrespective of the aging condition (The *p*-values for the immediate and aged values comparing the system with the chemical-cured adhesive to the system with the dual-cured adhesive were found to be *p* = 0.67 and *p* = 0.36, respectively). In the proximal root immediate group, all tested systems demonstrated comparable bond strength values (*p* = 0.051). However, following aging, the system utilizing the light-cured adhesive showed significantly lower values compared to the other two systems (The *p*-values for the comparison between the system with the light-cured adhesive and the system with the chemical-cured adhesive, as well as the dual-cured adhesive, were found to be *p* = 0.002 and *p* = 0.006, respectively), followed by the system with the chemical-cured adhesive. Among the proximal root dentin aged group, the system with the dual-cured adhesive exhibited the highest bond strength values.

Aging showed a negative effect on all tested restorative systems, regardless of the regional dentin type. Furthermore, the systems employing light-cured and chemical-cured adhesives were significantly affected by regional dentin variation after aging (*p* < 0.05). The system with the dual-cured adhesive was the only one that showed no statistical differences in regional dentin variations, regardless of the aging process (The *p*-values for the immediate measurement (*p* = 0.46) and after aging (*p* = 0.67) were obtained).

### Failure patterns

Table 3 illustrates the distribution of failure modes and pre-test failures across all subgroups presented in percentages. A notable interaction was observed between failure patterns and aging (*p* = 0.014). However, there were no significant interactions between failure patterns and restorative system or regional dentin type (*p* = 0.34, *p* = 0.39). Irrespective of the subgroup analyzed, adhesive failure patterns were predominantly observed followed by mixed patterns. Analyzing the pre-test failure percentages concerning the restorative system variable using one-way ANOVA did not reveal any significant differences (*p* = 0.54). Furthermore, the independent t-test indicated no statistically significant variance in pre-test failure rates concerning aging and regional dentin variables (*p* = 0.18, *p* = 0.26).

### Nanoleakage results

**Fig. 2:**
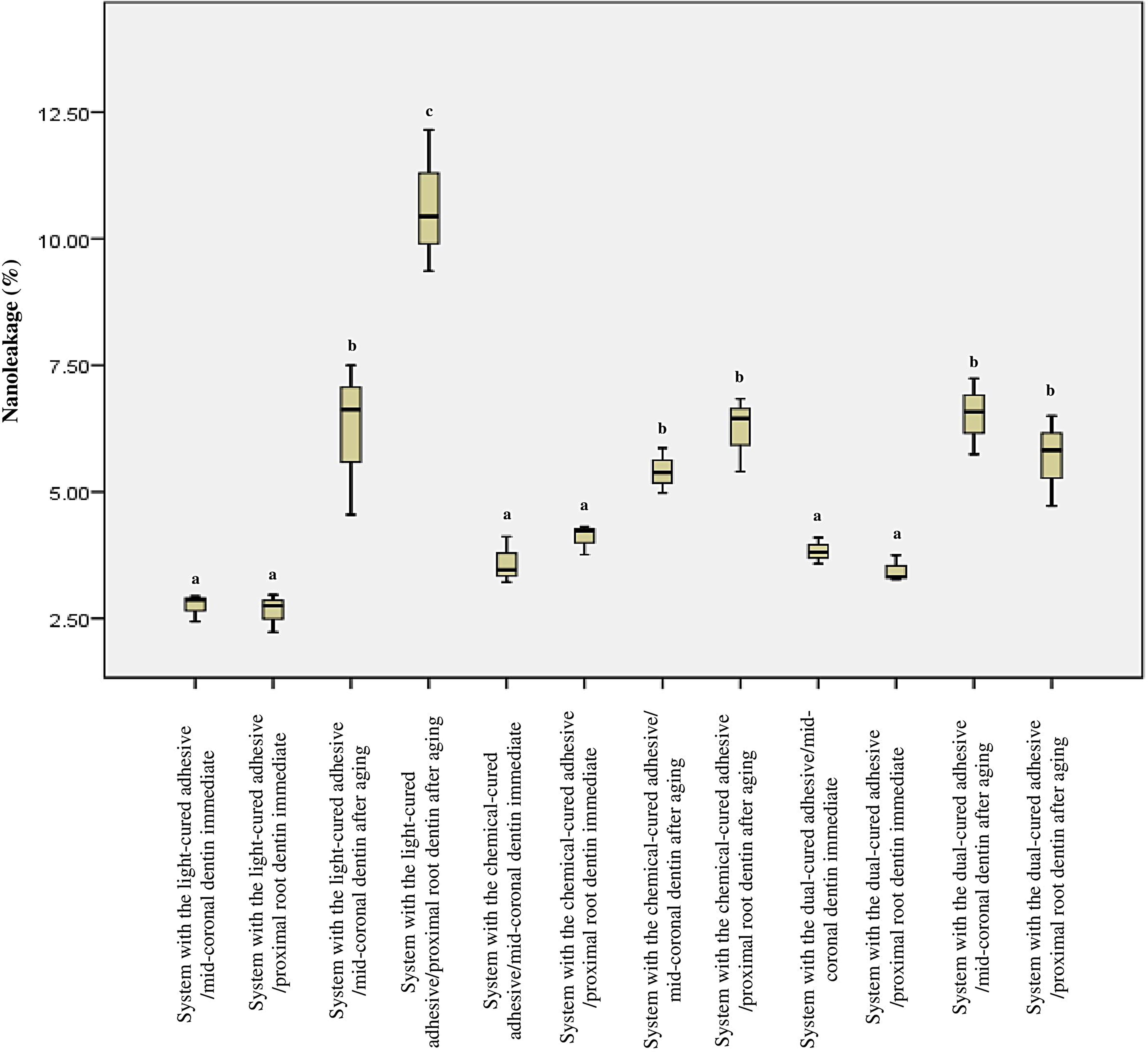
Box plots depicting the nanoleakage results for all the tested restorative systems with both mid-coronal dentin and proximal root dentin, immediately and after aging. In cases where the lowercase letters are the same, there was no significant difference between them (*p* < 0.05)

**Fig. 3:**
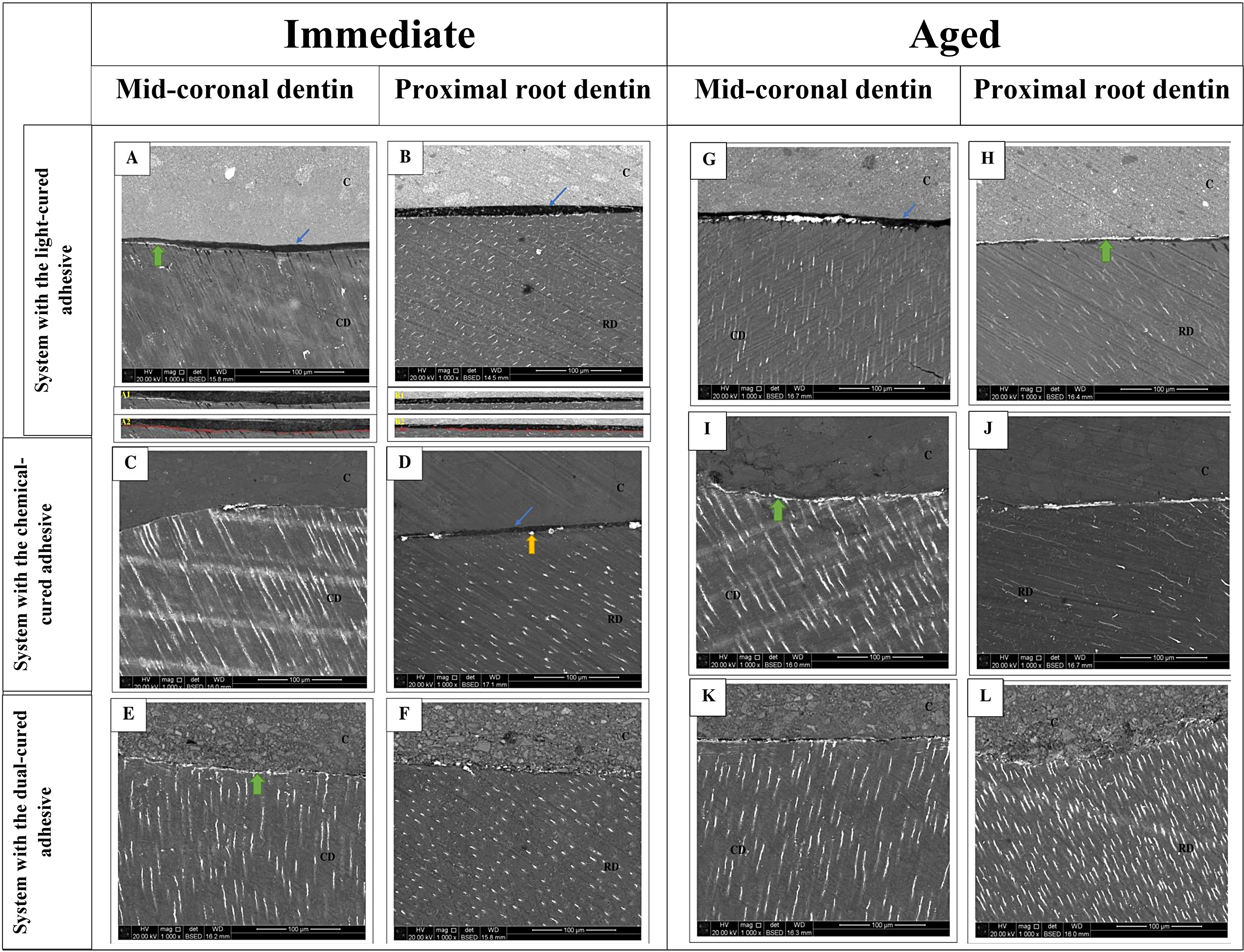
Representative backscattered SEM images were captured at a magnification of 1,000× to observe the nanoleakage in the immediate (**A**-**F**) and aged (**G**-**L**) subgroups. In **A** and **B**, the adhesive layer is indicated by a blue arrow, and the reticular nanoleakage pattern is represented by a green arrow. In **C** and **D**, the adhesive layer is indicated by a blue arrow, and the spotted nanoleakage pattern is represented by a yellow arrow. In **E**, the reticular nanoleakage pattern is indicated by a green arrow. **A1** and **B1** represent the selected areas of analysis. **A2** and **B2** are marked images showing the percentage of silver particle penetration as red pixels at the resin/mid-coronal dentin-proximal root dentin interfaces, using digital image analysis software. For the aged subgroups: in **G** and **H**, the adhesive layer is indicated by a blue arrow, and the reticular nanoleakage pattern is represented by a green arrow. In **I**, the reticular nanoleakage pattern is indicated by a green arrow. Abbreviations used are: **C** for composite resin, **CD** for mid-coronal dentin, and **RD** for proximal root dentin

Figure 2 illustrates the nanoleakage percentages of all the studied groups under both aging conditions. The Kruskal-Wallis test revealed that only the aging variable had a significant effect on the nanoleakage results (*p* < 0.001), while neither the restorative system type nor the regional dentin type had a significant effect (*p* = 0.89 and *p* = 0.68, respectively). The immediate subgroups exhibited significantly lower nanoleakage values compared to the aged subgroups (*p* < 0.05) regardless of the restorative system or regional dentin type. Within each aging condition separately, neither the restorative system nor the regional dentin type had an effect on the nanoleakage results, except for the restorative system with the light-cured adhesive/gingival dentin interface group after aging, which showed the highest statistically significant nanoleakage values. Figure 3 presents representative nanoleakage results of the tested groups displaying both immediate and post-aging conditions. The Spearman correlation coefficient revealed a moderately significant negative association between the mean µTBS and the nanoleakage data (ρ = -0.683, *p* < 0.001).

### Elemental analysis results

**Fig. 4:**
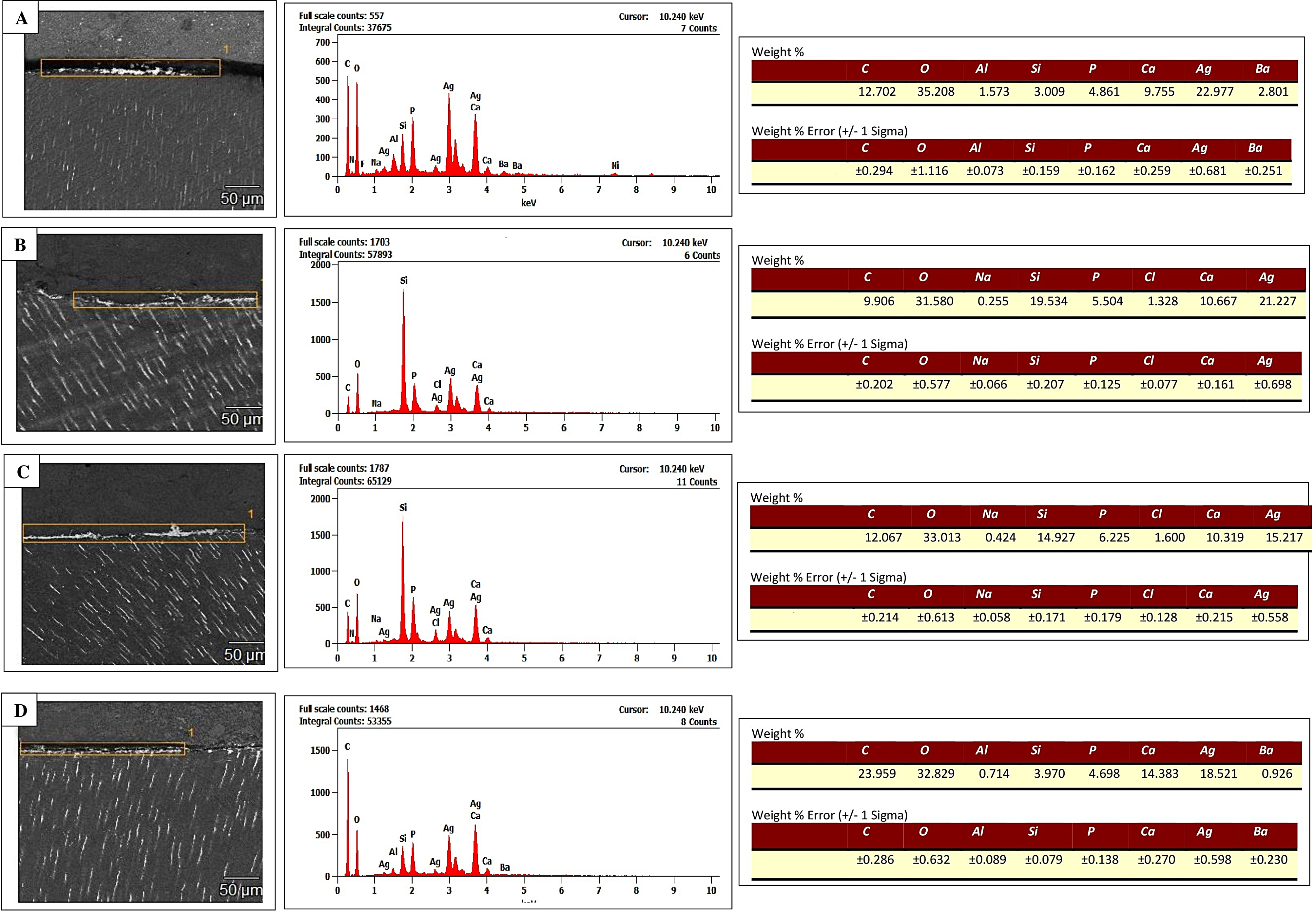
SEM micrographs and weight percentages (wt%) of elements obtained for various restorative systems at the interfaces of mid-coronal and proximal root dentin. **A** shows the interface between the system with the light-cured adhesive and mid-coronal dentin after aging, and **B** displays the immediate interface between the system with the chemical-cured adhesive and mid-coronal dentin. **C** represents the immediate interface between the system with the chemical-cured adhesive and proximal root dentin, and **D** illustrates the immediate interface between the system with the dual-cured adhesive and mid-coronal dentin. Notably, distinct silver peaks were observed in the elemental energy spectra. A map scan, indicated by a yellow rectangle within each image, revealed the presence of metallic silver particles

**Fig. 5:**
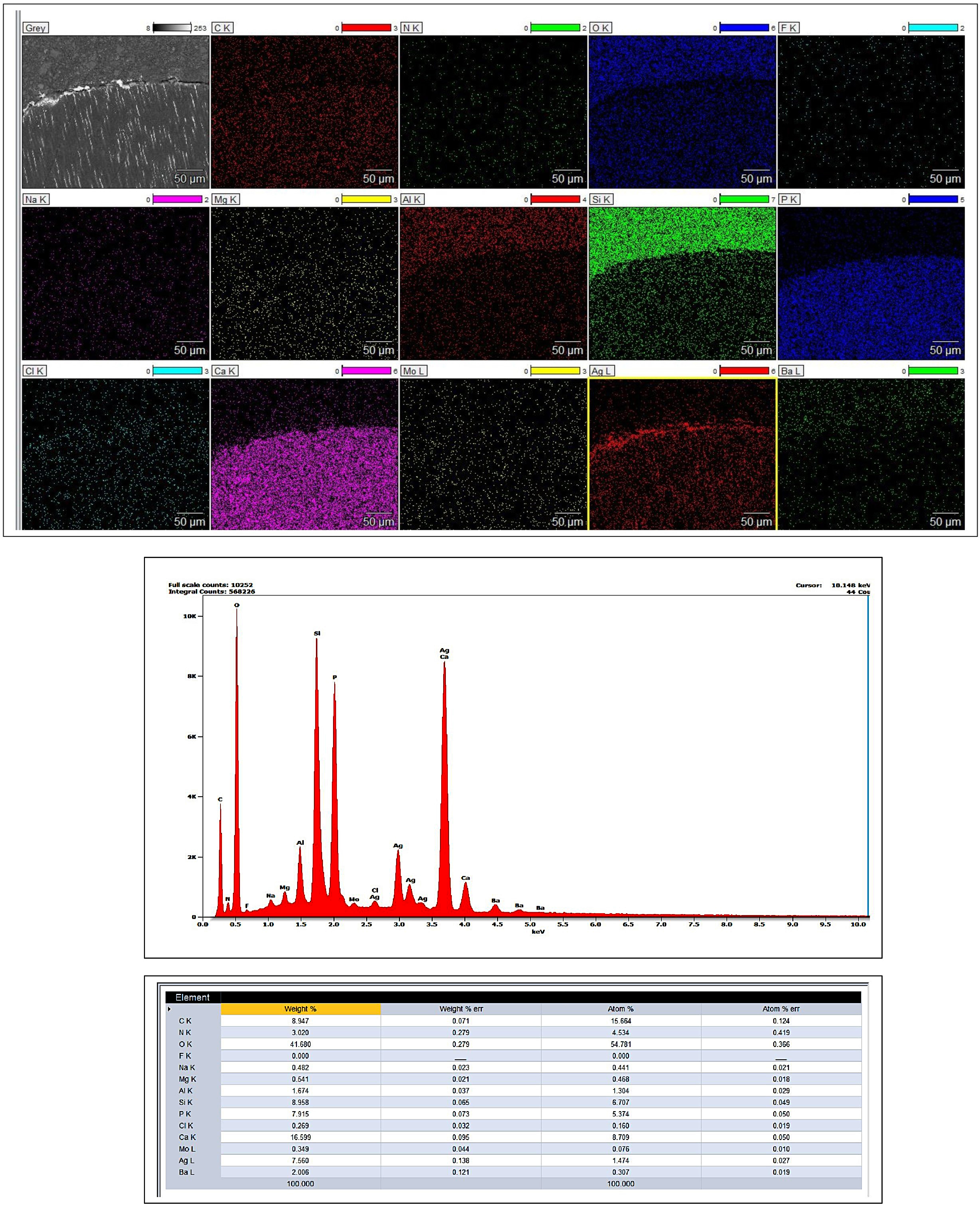
EDX mapping performed on a representative system with the dual-cured adhesive /mid-coronal dentin aged interface, revealing distinct elemental compositions for each component. The resin composite exhibited a higher concentration of silicon (Si), while the interface showed a higher concentration of silver (Ag), and the mid-coronal dentin displayed a higher concentration of calcium (Ca). The lower part of the image graphically represents the weight percentages (wt%) of the elements found in the entire scanned area

Figures 4 and 5 display SEM/EDS images and mappings of the interfaces combined with graphical representations indicating the weight% (wt%) of elements. The EDS analysis and mapping effectively identified the presence of elemental silver at specific locations, thereby providing supporting evidence for the observed nanoleakage results.

### Degree of conversion results

**Fig. 6:**
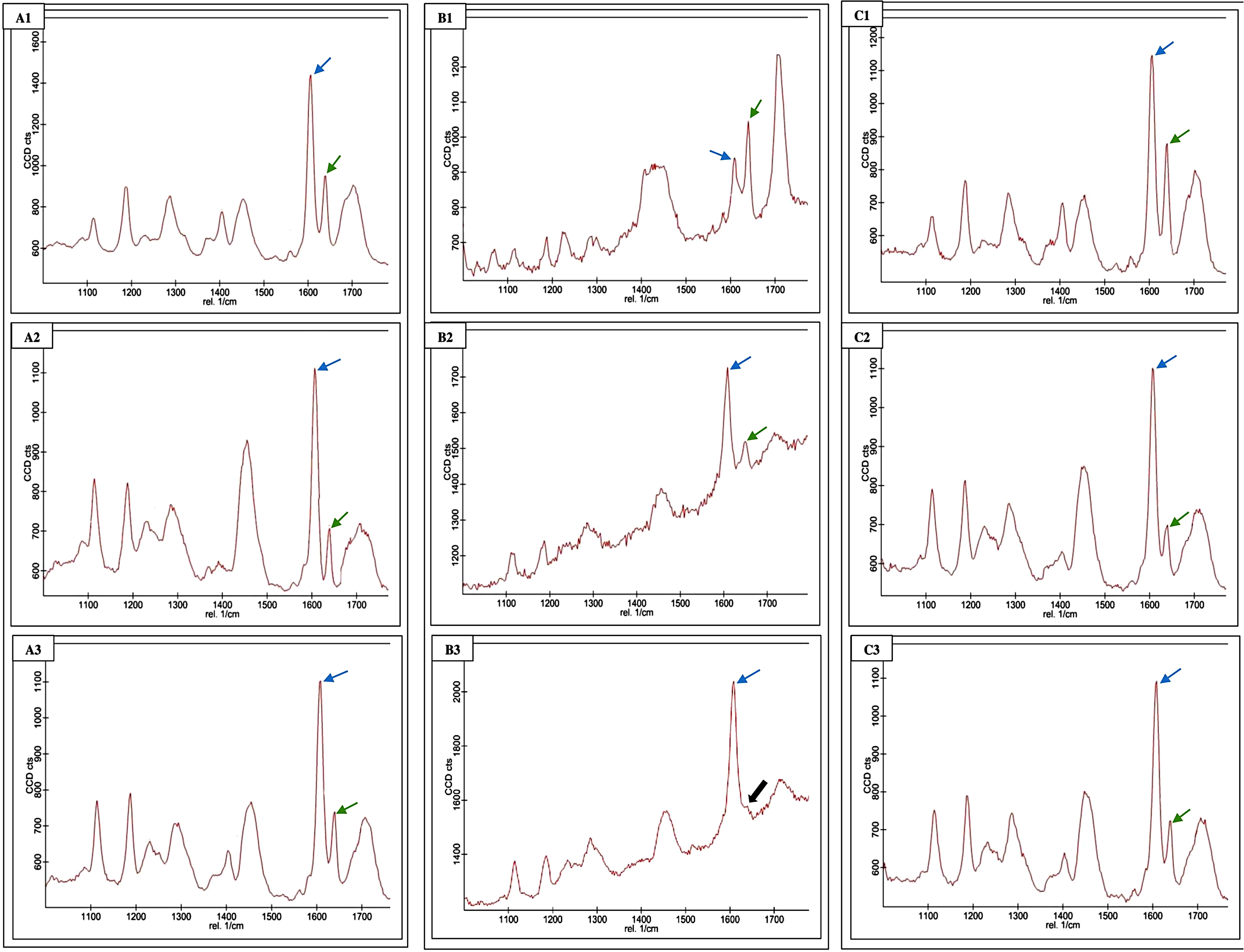
The spectra of uncured and polymerized adhesives were examined, with different distances considered for the light-cured and dual-cured adhesives. The reference peak is 1609 cm^− 1^ (blue arrows), and the reactive peak at 1639 cm^− 1^ (green arrows). In the case of the light-cured adhesive (**A)**, **A1** represented the uncured state, **A2** denoted the adhesive cured at 3 mm, and **A3** represented the adhesive cured at 7 mm. For the chemical-cured adhesive (**B)**, **B1** represented the uncured state, while **B2** and **B3** indicated the cured adhesive (the black arrow in **B3** refers to the missed 1639 cm^− 1^ peak). Similarly, for the dual-cured adhesive **(C)**, **C1** represented the uncured state, **C2** indicated the cured adhesive at 3 mm, and **C3** indicated the cured adhesive at 7 mm

Table 4 presents the degree of conversion results for all tested adhesives considering both curing distances (3 and 7 mm) for light-cured and dual-cured adhesives. Moreover, Fig. 6 illustrates representative spectra of uncured and polymerized adhesives at various distances.

**Table 4:** The mean degree of conversion percentages ± standard deviation for the three adhesives tested, assessed at both 3 and 7 mm curing distances for the light-cured and dual-cured adhesives

Adhesive tested	Degree of conversion
Light-cured adhesive at 3 mm	84.66 ± 6.57^a^
Light-cured adhesive at 7 mm	52.08 ± 7.20^b^
Chemical-cured adhesive	81.48 ± 5.64^a^
Dual-cured adhesive at 3 mm	78.55 ± 7.32^a^
Dual-cured adhesive at 7 mm	75.79 ± 6.47^a^

Groups in identified with the same superscripted lower case letters are not significantly different among each other. (*p* < 0.05)

A statistically significant difference was observed between the light-cured adhesive at a distance of 7 mm and the other groups (*p* < 0.5). However, no statistically significant differences were found among other adhesive groups (*p* = 0.26). During the evaluation of the chemical-cured adhesive, the peak at 1639 cm^− 1^ was undetectable in two specimens after polymerization (Fig. 6. B3, black arrow), those two specimens were replaced.

## Discussion

Although one of the main inquiries in the present study focused on the adhesive system, each group was subjected to testing the resin composite of the corresponding brand along with the adhesive. Standardizing the resin composite would have facilitated a fairer comparison among the adhesives as the sole variable under examination. However, utilizing the resin composite from the same brand as the adhesive ensured optimal outcomes for each adhesive being evaluated [11]. Manufacturers may have optimized the material properties of both the composite and adhesive to enhance their compatibility resulting in improved bonding and overall performance [25]. This could be a result of unique chemical compatibility between the two materials. Additionally, this ensures that the materials are used according to the manufacturer’s recommendations.

Moreover, all adhesives tested in the study were mild universal adhesives to ensure standardization considering the performance of universal adhesives that has been demonstrated to be influenced by their pH levels [6].

In the current study, universal adhesives were utilized in the self-etch mode. Perdigão [29] elucidated that the etching of dentin with phosphoric acid leads to the depletion of calcium from the dentin surface. In the absence of calcium, the mechanism by which functional monomers, particularly 10-MDP can form ionic bonds with etched dentin remains uncertain. A recent systematic review mentioned that self-etch adhesives or universal adhesives applied in self-etch or selective enamel etch mode offer advantages for deep margin elevation due to the risk of over-etching dentin with total-etch adhesives [30]. Half of the specimens in this study are considered deep proximal Class II cavities and bond to proximal root dentin. That is why the three tested universal adhesives were used in the self-etch mode. The heights of the opaque acrylic plastic rings (3 and 7 mm) were chosen based on their relevance to the distance to the pulpal floor and gingival step encountered in Class II cavity preparation during clinical situations [25]. While the µTBS test is commonly employed, concerns have been raised regarding its clinical significance, inconsistent outcomes in various studies, the absence of standardization, and the limited comprehension of the link between in vitro results and real clinical efficacy [31]. Even with these critiques, the µTBS test is still a key technique for assessing interfacial adhesive bonds [28].

Pre-test failures in this study were excluded from the statistical analysis. According to Scherrer et al. [32]. incorporating pre-test failures into the statistical analysis could potentially introduce data scattering. The bond strength in the current study was tested while bonding to flat dentin. Although the use of a high C-factor cavity is used to simulate the actual stresses experienced by the tested restorative systems [33], the cavity preparation performed by an operator may lack standardization and strict flattening of the dentin surface, which is necessary for a sensitive test like µTBS when compared to bonding to strictly flat surfaces created by the ISOMET. The smear layer is expected to possess different properties due to variations in pressure, cutting techniques, and cooling efficiency [33]. These differences in smear layer characteristics combined with the use of mild universal adhesives might impact the results. The decision to test only four beams within each specimen for the proximal root dentin group was based on a previous pilot study aiming to approximate the number of beams that could be obtained solely from the proximal dentin.

Nanoleakage refers to the flaws at the resin-dentin junction that could act as channels for the deterioration of resin/dentin interfaces over time [34]. These imperfections arise when resin does not penetrate the nanoscale gaps surrounding exposed collagen fibrils or when residual moisture persists in those regions due to incomplete displacement by adhesive or inadequate monomer conversion [34]. According to Van Meerbeek et al. [35]. nanoleakage is considered the primary mechanism for bond degradation in dentin.

In the current study, nanoleakage was assessed in Class II cavities instead of using separate specimens for different regional dentin. This approach was selected to ensure standardized curing and testing conditions for both regional dentin areas. Although it would have been more valuable to obtain bond strength data and nanoleakage measurements using the same specimens, the number of available beams from the proximal root dentin specimens was limited. In this study, only three images were taken for each regional area interface of each specimen (A total of 9 images per interface for each subgroup within each aging condition) [36, 37], which were evaluated for nanoleakage. Capturing the entire resin/dentin interface for mid-coronal dentin and proximal root dentin at a magnification of ×1,000 would require a considerable number of images. Analyzing such a large number of images could pose challenges. EDS analysis is a reliable and precise method for both quantitative and qualitative examination of element distribution [38]. It is known for its sensitivity and accuracy in detecting chemical components [38]. By employing EDS, the presence of elemental silver at specific locations could be definitively verified [39]. This approach helped to avoid potential misinterpretation of brightness caused solely by the electron microscope edge effect, preventing any misleading conclusions about the presence of silver particles [40].

Numerous research studies suggested that Raman spectroscopy is a practical and precise method for evaluating how different factors influence the degree of conversion in resin composites, especially when contrasted with Fourier transform infrared spectroscopy [36]. While assessing the degree of conversion within the hybrid layer of adhesive resins would have been more informative, this evaluation was carried out after the µTBS and nanoleakage results. The purpose was to obtain additional insights into the contradictory behavior of the tested system with the light-cured adhesive. Despite exhibiting the highest bond strength values at the mid-coronal regardless of the aging condition, it showed the lowest bond strength and the highest nanoleakage values at the proximal root dentin after aging. If an in-situ degree of conversion measurement had been conducted in this study, the factor of regional dentin type would still be present. Therefore, assessing the degree of conversion on the glass slide only allowed testing the influence of the curing distance on the degree of conversion of the adhesives. However, further investigation is necessary in the future, considering that regional dentin was found to be a significant factor affecting the bond strength results based on the current study’s findings.

Based on the study findings, the restorative system type did not have a significant influence on either the bond strength or nanoleakage results. Therefore, the first null hypothesis was accepted. However, the aging process was found to have a significant effect on both the bond strength and nanoleakage results. As a result, the second null hypothesis was rejected.

The deterioration of bond strength observed in all subgroups after aging can be attributed to two main factors: the deterioration of the hybrid layer and the mechanical stress resulting from the differing thermal expansion coefficients of the tooth and the restoration [7]. Although the universal adhesives used in this study have different ingredient compositions, they all share a common essential component: water. Previous reports have explained that residual water, which remains after adhesive evaporation, can lead to hydrolysis of polymeric resins and enzymatic degradation of collagen fibrils [19]. Long-term water storage experiments have shown that degradation is accelerated by the hydrolysis of hydrophilic resin components present in the adhesives [19]. The light-cured and dual-cured adhesives tested in this study are simplified adhesives with hydrophilic properties. They function as semi-permeable membranes making them more prone to water absorption. This increased water absorption can expedite the deterioration and hydrolysis of components at the interface [25]. The chemical-cured adhesive examined in the study contains a notable concentration of Hydroxyethyl Methacrylate (HEMA) (10–30%) and acetone, which have the potential to enhance water sorption and subsequent hydrolysis [25]. This observation also explains the sole significant effect of aging observed in the failure mode analysis, wherein no cohesive failures were observed in any of the aged subgroups.

The study findings indicated that the bond strength values were influenced by regional variations in dentin, especially after aging. However, the nanoleakage results were not affected by this variable. As a result, the third null hypothesis was partially rejected. Dentin tubules, which extend from the dentin-enamel junction to the inner pulp chamber, exhibit variations in density and alignment across different regions of the tooth [41]. These variations result in differences in the amount of dentin between the tubules and the orientation of the tubules themselves based on the location of the tooth [42].

Occlusal dentin has perpendicular dentinal tubules, while gingival dentin has parallel tubules leading to a larger amount of peritubular dentin in the gingival region [42]. Consequently, there is a smaller area of intertubular dentin available for the formation of the hybrid layer at the gingival wall [42]. However, a previous study suggested that self-etching systems are less influenced by regional dentin variations due to their ability to dissolve smear plugs, reduce dentin permeability, and minimize sensitivity to water pressure [16]. There is limited data available on the effects of different regional dentin variability on bond strength using universal adhesives. Moreover, the existing literature on this topic is outdated and based on older generations of adhesives, which may not perfectly align with the testing setup used in this study.

It is worth mentioning that the distribution of the pre-test failure data was not affected by any of the variables in the study. The failures were evenly distributed across different groups and were not concentrated in any specific subgroup. This suggests that the occurrence of these pre-test failures may be attributed to random preparation problems [43].

Irrespective of the aging process, the restorative system utilizing the light-cured adhesive demonstrated the highest bond strength and exhibited the lowest nanoleakage values on mid-coronal dentin. This outcome can be attributed to the composition of the adhesive, which possesses a mildly acidic nature that leads to partial demineralization of the dentin. Consequently, hydroxyapatite crystals are left around the collagen fibrils. Additionally, the adhesive contains 10-methacryloyloxydecyl dihydrogen phosphate (MDP), which forms a chemical bond with the dentin. The interaction between MDP and hydroxyapatite produces a stable nano-layer that enhances the strength of the adhesive interface [36]. This phenomenon of MDP-Ca salt deposition and nano-layering elucidates the remarkable bond stability observed in laboratory and clinical research [36].

The adhesives used in the other two restorative systems do not contain 10-MDP. In the system utilizing the chemically cured adhesive, a 3D-SR monomer is present, which forms a chemical bond with the tooth through interactions at multiple points with the dentin. Furthermore, this monomer facilitates three-dimensional crosslinking, enhancing the bond strength [25]. The tested dual-cured adhesive contains phosphorylated methacrylate, which has similar chemical activity to 10-MDP [25]. However, based on the results of mid-coronal dentin, the adhesive system containing 10-MDP demonstrates better durability at the adhesive interface compared to the other two.

While there were no statistically significant differences in proximal root dentin bond strength and nanoleakage values among all the tested restorative systems immediately, notable variations were observed after the aging process. Specifically, the restorative system utilizing the light-cured adhesive displayed significantly lower bond strength and higher nanoleakage values compared to the other two systems following aging, despite demonstrating high bond strength values in the mid-coronal dentin category within the same system. This inconsistency can be attributed to the considerably lower degree of conversion in the tested light-cured adhesive at a distance of 7 mm. Inadequate polymerization of the adhesive can lead to the formation of a porous zone, which may initiate cracks and subsequently result in bonding failure [37, 44]. This is evidenced by the reduced bond strength and increased nanoleakage observed in such cases.

In a previous study [9], the effect of increased curing distance on the degree of conversion and shear bond strength values was highlighted. The researchers noted that even a slight change in the degree of polymerization conversion of the adhesive (and possibly the initial thin layer of composite) has a significant effect on the mechanical properties and longevity of the interfaces between the tooth and adhesive, as well as the adhesive and composite, particularly in a moist oral environment. These findings may support the results of the current study, which observed a notable decrease in bond strength values and an increase in nanoleakage when the light-cured adhesive system was cured at a distance of 7 mm from the light-curing device, compared to a distance of 3 mm. Indeed, Rode et al. [21]. emphasized the importance of maintaining a distance of no more than 3 mm between the LED light curing tip and the surface of the resinous material to achieve an adequate degree of conversion.

After aging, the bond strength values of the system using the chemical-cured adhesive on the proximal root dentin floor were significantly lower compared to the system using the dual-cured adhesive. This difference may be attributed to the variation in solvent type. The chemical-cured adhesive contains acetone solvent, and previous reports have indicated that higher acetone content can lead to thinner adhesive layers and reduce dentin µTBS [44, 45]. This could have a negative effect when bonding to the challenging gingival dentin area.

Although different resin composites with varying filler volume ratios are used, which could indicate different volumetric shrinkage and polymerization shrinkage stresses [46], a previous study reported no statistically significant difference in µTBS when bonding different resin composites to flat dentin [33], similar to the approach used in our present study. Additionally, Almeida Junior et al. [47]. found a weak correlation between shrinkage volume and bond strength. Hence, our interpretation of the results focuses more on the variations in the adhesives employed.

The results of the current study revealed a moderate negative significant correlation between the bond strength and nanoleakage values, thus the fourth null hypothesis was rejected. This finding aligns with previous studies that have also reported a similar correlation [44, 48].

The results of the current study raise concerns regarding the use of restorative systems with light-cured adhesives in areas where the curing distance from the light-curing devices is extended, such as deep Class II gingival margins. These concerns are related to bond strength, nanoleakage, and degree of conversion. However, it is important to note that these results are specific to the tested restorative systems and cannot be generalized to other systems with light-cured adhesives. Additionally, it is recommended to include a positive control group consisting of dual-cured composites with either dual or chemical-cured adhesives. This control group will exclude the potential effect of curing distance from light-cured composites, which may affect the results. The recommendation to enhance the light-cured adhesive used in our study involves incorporating a touch-cure mechanism by adding a highly active chemical polymerization initiator to the light-curable adhesive formulation. This curing mode, commonly referred to as touch-cure, has been introduced to facilitate the bonding of dual-cured composites to dentin in areas with limited or no exposure to activating light [49]. The touch-cure mechanism has been proven to achieve not only improved but also faster polymerization [49]. Incorporating this touch-cure property into a light-curable adhesive enables polymerization initiation at the actual interface with dentin [50]. This provides a viable alternative for sealing the dentin interface without the need for separate light-curing of the adhesive [50]. Furthermore, testing the same hypothesis of the current study with longer aging periods is recommended. It is crucial to understand that the results of the current study do not imply an absolute restriction on the use of light-cured adhesives in cases with extended curing distances. However, detailed instructions from the adhesive manufacturer should be provided in these clinical situations.

## Conclusions

1. Aging has a detrimental effect on the bond durability of all tested restorative systems, regardless of the regional dentin variable. However, the regional dentin type only influences the bond strength and not the nanoleakage data of the tested restorative systems.
2. The bonding properties including bond strength, nanoleakage, and degree of conversion, of the tested system with the light-cured adhesive were adversely affected when the curing distance was increased from 3 to 7 mm after aging.

## Data Availability

The datasets generated and/or analyzed during the current study are not publicly available but are available from the corresponding author upon reasonable request.

## Declarations

### Clinical trial number

Not applicable. This study is a laboratory-based investigation and does not involve a clinical trial. Therefore, no clinical trial number has been assigned.
